# A Systematic Approach to Identify Markers of Distinctly Activated Human Macrophages

**DOI:** 10.3389/fimmu.2015.00253

**Published:** 2015-05-27

**Authors:** Bayan Sudan, Mark A. Wacker, Mary E. Wilson, Joel W. Graff

**Affiliations:** ^1^Infectious Diseases Division, Department of Internal Medicine, University of Iowa, Iowa City, IA, USA; ^2^Iowa City VA Medical Center, Iowa City, IA, USA

**Keywords:** human macrophages, activation markers, microarray, integrated fluidic circuit RT-PCR, macrophage polarization

## Abstract

Polarization has been a useful concept for describing activated macrophage phenotypes and gene expression profiles. However, macrophage activation status within tumors and other settings are often inferred based on only a few markers. Complicating matters for relevance to human biology, many macrophage activation markers have been best characterized in mice and sometimes are not similarly regulated in human macrophages. To identify novel markers of activated human macrophages, gene expression profiles for human macrophages of a single donor subjected to 33 distinct activating conditions were obtained and a set of putative activation markers were subsequently evaluated in macrophages from multiple donors using integrated fluidic circuit (IFC)-based RT-PCR. Using unsupervised hierarchical clustering of the microarray screen, highly altered transcripts (>4-fold change in expression) sorted the macrophage transcription profiles into two major and 13 minor clusters. Among the 1874 highly altered transcripts, over 100 were uniquely altered in one major or two related minor clusters. IFC PCR-derived data confirmed the microarray results and determined the kinetics of expression of potential macrophage activation markers. Transcripts encoding chemokines, cytokines, and cell surface were prominent in our analyses. The activation markers identified by this study could be used to better characterize tumor-associated macrophages from biopsies as well as other macrophage populations collected from human clinical samples.

## Introduction

Macrophages assume critical roles in almost every tissue and disease state through their ability to assume distinct functional capacities in different microenvironments. Macrophages respond to a variety of external stimuli to assume different polarized activation states. Distinctly polarized macrophages, modeled *in vitro* using specific activating conditions, can be defined by functional attributes such as microbicidal activity, and by unique gene expression profiles. An early study contrasting functional and gene expression differences between IFNγ- and IL-4-treated macrophages proposed that the latter phenotype be described as alternative activation (1), a very different macrophage phenotype from IFNγ- or classically activated macrophages. Since that time, many additional polarized macrophage types, induced by different stimuli, have been proposed.

Several competing systems have been proposed in an attempt to provide a framework that describes the complexity of macrophage polarization. The first system describes macrophage phenotypes as a linear continuum with M1 (classically activated) and M2 (alternatively activated) macrophages at opposite ends (2, 3). The second system describes macrophage phenotypes as a spectrum akin to a color wheel, with classically activated, wound healing, and regulatory macrophages used as examples of unique polarized phenotypes that do not fit well within a linear continuum (4). A modified version of the M1–M2 system acknowledged the diversity of macrophage phenotypes with descriptions such as M1a, M1b, M2a, M2b, and M2c (5, 6). Additions to the M1–M2 nomenclature system have proposed naming macrophages differentiated in the presence of CXCL4 as “M4” (7) and IL-17-treated macrophages “M17” (8). To standardize the burgeoning descriptions of polarized macrophage types, it has been suggested that the activation condition be defined in the name of the polarized macrophage [M(IL-4), M(IL-10), M(LPS), M(IFNγ), and so forth (9)]. To preserve clarity, we have employed this descriptive nomenclature system to describe the activated macrophages in the current report (Table 1).

**Table 1:** **Macrophage-activating conditions and nomenclature used in this study**.

Single stimulus treatments	Previous nomenclature	Current nomenclature
1. GM-CSF (100 ng/ml)	M1	M(GM-CSF)
2. IFNβ (20 ng/ml)		M(IFNβ)
3. IFNγ (20 ng/ml)	M1, classical	M(IFNγ)
4. IL-1β (100 ng/ml)		M(IL-1β)
5. IL-4 (20 ng/ml)	M2, M2a, alternative, wound healing	M(IL-4)
6. IL-10 (50 ng/ml)	M2c	M(IL-10)
7. TGFβ (5 ng/ml)	M2c	M(TGFβ)
8. TNFα (100 ng/ml)		M(TNFα)
9. Curdlan (20 μg/ml)^a^		M(Curdlan)
10. TDB (20 μg/ml)		M(TDB)
11. PolyI:C (2 μg/ml)		M(PolyI:C)
12. LPS (10 ng/ml)	M1, classical	M(LPS10)
13. LPS (100 ng/ml)	M1, classical	M(LPS100)
14. Adenosine (100 μM)		M(Ado)
15. IgG-OVA immune complexes (IC)^a^		M(IC)
16. Dexamethasone (100 nM)	M2c	M(Dex)

**Combinational treatments**	**Previous nomenclature**	**Current nomenclature**

17. TDB + IFNγ		M(TDB + IFNγ)
18. TDB + IC^a^		M(TDB + IC)
19. TDB + IL-4		M(TDB + IL-4)
20. TDB + IL-10		M(TDB + IL-10)
21. LPS (10 ng/ml) + IFNγ	M1	M(LPS + IFNγ)
22. LPS (100 ng/ml) + IC^a^	M2b, regulatory	M(LPS + IC)
23. LPS (10 ng/ml) + IL-4		M(LPS + IL-4)
24. LPS (10 ng/ml) + IL-10		M(LPS + IL-10)
25. Adenosine + IFNγ		M(Ado + IFNγ)
26. Adenosine + IC^a^		M(Ado + IC)
27. Adenosine + IL-10		M(Ado + IL-10)
28. TGFβ + GM-CSF		M(TGFβ + GM-CSF)
29. TGFβ + IL-1β		M(TGFβ + IL-1β)
30. TGFβ + LPS (100 ng/ml)		M(LPS + TGFβ)
31. Dexamethasone + GM-CSF		M(Dex + GM-CSF)
32. Dexamethasone + IL-1β		M(Dex + IL-1β)
33. Dexamethasone + LPS (100 ng/ml)		M(LPS + Dex)

*^a^Treatments with chicken ovalbumin and with Curdlan likely had endotoxin contamination due to the extraction processes used to obtain these reagents (10, 11)*.

Macrophages are often very abundant within tumors (12, 13). There is evidence that macrophages can promote tumorigenesis, tumor growth, and metastasis (14). Despite macrophage pro-tumor activities, tumor-associated macrophages (TAMs) display a wide range of phenotypic diversity within a tumor due to ontogeny, activation signals, and localization (15). The plasticity of macrophage phenotypes is well known (16, 17) and this characteristic has provided a therapeutic target whereby macrophages are encouraged to switch functionally from pro-tumor to anti-tumor. Clinical approaches that modify macrophage activation in this way include blockade of M-CSF, low-dose irradiation, and combinational therapies (18–21). What is lacking is a thoroughly characterized and reliable set of macrophage activation markers that would allow for improved characterization of activation patterns, and monitoring of the therapeutic efficacy of macrophage-targeted treatments.

Gene expression profiles using microarrays have been used to analyze activation of primary human monocytes and monocyte-derived macrophages (MDMs) (7, 22–32). Until very recently (33), most transcriptome-based approaches to characterize polarized macrophages contrasted two macrophage-activating conditions in each study. Using a blood sample from a single human donor, we surveyed gene expression profiles in primary macrophages activated with 33 different activating conditions. This data set served as a rich resource for identifying putative human macrophage activation markers. As a follow-up approach, integrated fluidic circuit (IFC)-based RT-PCR was used to examine a panel of transcripts to verify the reproducibility of the gene expression changes from multiple donors. This latter assay was also used to determine the expression kinetics of previously described markers of human macrophages as well as novel markers identified by the microarray-based screen.

## Materials and Methods

### Human subjects

Human subject protocols were approved by Institutional Review Boards of the University of Iowa and the Iowa City Veterans Affairs Medical Center. Peripheral blood samples from anonymous, healthy donors were acquired through the DeGowin Blood Bank at the University of Iowa.

### Integrated fluidic circuit-based RT-PCR

RNA purified from MDMs using TRIzol was reverse transcribed in random hexamer-primed reactions with SuperScript III RT (Invitrogen). The cDNA was pre-amplified for 14 PCR cycles in reactions primed by a master mix of 48 TaqMan Gene Expression Assays (Applied Biosystems) using PreAmp Master Mix (Applied Biosystems) with a modified protocol according to recommendations by Fluidigm. Following a 1:5 dilution of pre-amplified product in water, 48 samples and 48 TaqMan Gene Expression Assays were loaded onto 48.48 Dynamic Array IFC plates (Fluidigm) using the 48.48 MX IFC Controller (Fluidigm). Real-time PCR was performed using the BioMark System for Genetic Analysis (Fluidigm). Cycle threshold (Ct) values were determined using real-time PCR analysis v3.1.3 software (Fluidigm). Ct values corresponding to transcripts encoding ACTB, B2M, and TBP were used as endogenous controls. Changes in transcript expression were calculated using the ΔΔCt method and converted to log_2_ scale using Excel 2010. Line graphs of time course experiments were generated using Prism 6 (GraphPad). Heat maps were generated using Partek Genomic Suite software.

### Cell purification and culture

Peripheral blood mononuclear cells (PBMCs) were isolated from the blood by density sedimentation using Ficoll-Paque PLUS (GE Healthcare) and maintained in Petri dishes at a density of 5e7 cells/dish in 10 ml RP-10 medium [RPMI 1640 medium (Gibco) supplemented with 10% fetal bovine serum (Gibco), 100 U/ml penicillin, 50 μg/ml gentamicin, and 5 ng/ml M-CSF (eBioscience)]. After 10 days, non-adherent cells were removed by rinsing and the adherent MDMs were dislodged with cell scraping following incubation at 37°C for 10 min in 0.25% Trypsin/1 mM EDTA solution (Gibco). MDMs were seeded in 12-well tissue culture-treated plates (Corning) at 5e5 cells/well in 2 ml RP-10 and allowed to rest for 2 days at 37°C. Before treatment with macrophage-activating conditions, the culture medium was replaced with 1 ml fresh RP-10 per well. At 24 h post-treatment, RNA was purified from MDMs using TRIzol Reagent (Invitrogen).

### Macrophage-activating stimuli

All stock solutions were stored at −80°C unless otherwise noted. The sources of human recombinant cytokines were as follows: IL-1β (eBioscience), IL-4 (PeproTech), IL-10 (R&D Systems), IFNβ (PeproTech), IFNγ (PeproTech), GM-CSF (eBioscience), TNFα (PeproTech), and TGFβ (R&D Systems). These cytokines were stored at concentrations recommended by the manufacturers and were subjected to no more than two freeze–thaw cycles. Dexamethasone powder (Sigma-Aldrich) was suspended in 1 part ethanol and subsequently diluted in 49 parts medium to a stock concentration of 50 μM. Phenol-extracted *Escherichia coli* 055:B5 LPS (Sigma-Aldrich) and polyinosinic:polycytidylic acid sodium salt (PolyI:C) (Sigma-Aldrich) were stored at a stock concentration of 1 mg/ml in RP-10. Adenosine (Sigma-Aldrich) was suspended in RP-10 at a stock concentration of 10 mM.

Chicken ovalbumin (MP Biomedicals) was suspended at 2 mg/ml in PBS lacking Ca^++^ or Mg^++^ (Gibco) and goat anti-chicken ovalbumin (MP Biomedicals) was suspended in water at 16 mg/ml. Immune complexes (IC) were prepared fresh for each experiment by combining ~10:1M excess of antibody to antigen and incubating with end-over-end rotation at room temperature for 30 min. Curdlan (InvivoGen) was also freshly prepared for each experiment by suspension in RP-10 at a concentration of 1 mg/ml.

Trehalose-6,6-dibehenate (TDB) (InvivoGen) was suspended at a concentration of 10 mg/ml in DMSO and heated to 60°C for 30 s. After vortexing, the TDB/DMSO solution was diluted to 1 mg/ml by the addition of PBS. This stock solution was heated to 60°C for 15 min and stored at 4°C.

### Microarrays

RNA sample preparation for microarrays and the subsequent hybridization to the Illumina beadchips were performed at the University of Iowa DNA Facility. Three Human HT-12 v4 BeadChips (Illumina) were processed individually in this experiment with 1 sample from an untreated control and 11 samples from polarized macrophages loaded onto each array. Briefly, 100 ng total RNA from each of the 36 samples was amplified and converted to biotin-cRNA using the Epicenter TargetAmp-Nano Labeling Kit for Illumina Expression BeadChip (Illumina). The biotin-aRNA product was purified using the RNeasy MinElute Cleanup Kit (Qiagen) according to modifications from Epicenter. Seven hundred fifty nanograms of this product were mixed with Illumina hybridization buffer, placed onto each beadchip array, and incubated with rocking at 58°C for 17 h in an Illumina Hybridization Oven. Following hybridization, the arrays were washed, blocked, and stained with streptavidin-Cy3 using the Whole-Genome Gene Expression Direct Hybridization Assay (Illumina). Beadchip arrays were scanned with the iScan System (Illumina) and data were collected using the GenomeStudio software v2011.1 (Illumina). The expression data has been deposited in NCBI Geo repository (GSE68854).

### Transcript expression analysis

Partek Genomic Suite v6.5 (Partek) was used to perform robust multi-array averaging and to calculate gene expression changes. A data set comprising of 1874 transcripts with changes in expression of more than fourfold relative to untreated controls was submitted to unsupervised hierarchical clustering and principal components analysis using default settings in Partek Genomic Suite software. Briefly, for unsupervised hierarchical clustering, agglomerative clustering was used to determine Euclidean dissimilarity distances using an average linkage method. For principal components analysis, a dispersion matrix based on correlations was normalized using Eigenvector scaling. Contribution of individual transcripts to each of the principal components was determined using the FactoMineR package in R. After principal component analysis was completed the contribution of each transcript to each of the components was extracted and ranked using Excel 2010.

Correlation coefficients were calculated for each pairwise combination of the 33 activated macrophage expression profiles for the 1874 regulated transcripts using the corandPvalue function of the WGCNA package in R. The data were then converted to heat maps using Excel (Microsoft).

For gene ontology (GO) analysis, the STRING database (version 9.05; string-db.org) was used to identify the 1615 protein coding RNAs in our set of 1874 regulated transcripts. Also, within the STRING database website, the GO categories enriched in the set of 1615 regulated transcripts identified as protein coding RNAs were determined.

## Results

### Survey of proposed human macrophage activation marker expression in MDMs responding to six distinct activation conditions

**Figure 1:**
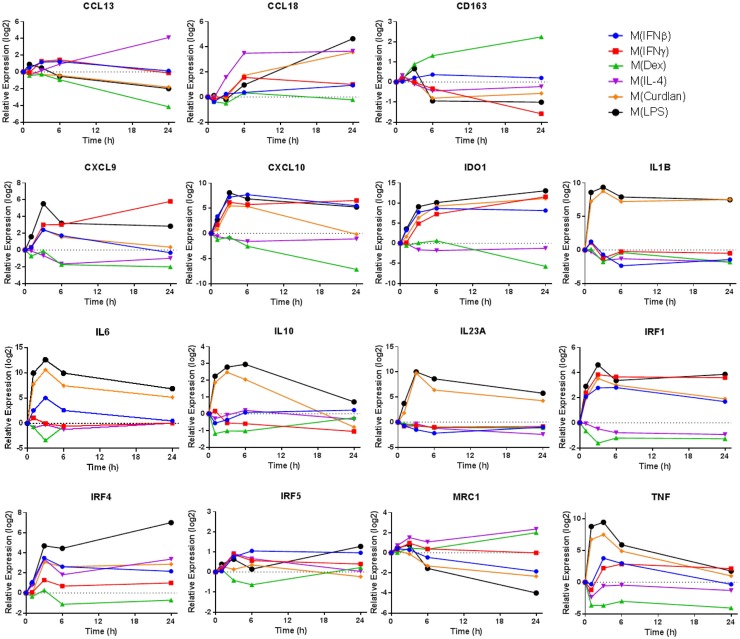
**Expression kinetics of previously proposed macrophage activation markers**. At 1, 3, 6, and 24 h time points, RNA was collected from untreated MDM controls as well as M(IFNβ), M(IFNγ), M(Dex), M(IL-4), M(Curdlan), and M(LPS). Using IFC-based RT-PCR, the changes in expression for each indicated transcript relative to the untreated MDM controls was determined at each time point for the six types of activated MDMs (*N* = 1).

Transcripts used as markers of polarized human macrophages should change expression in response to one stimulus or a limited number of related activation stimuli. Additionally, macrophage activation markers should have sustained, rather than transient, changes in expression. In primary human macrophages responding to a variety of activation conditions, we evaluated the expression kinetics of transcripts that encode 11 proposed activation markers (4, 6, 9) over a 24-h period (Figure 1). Several observations from this survey were notable. First, some commonly assessed transcripts, TNF and IL-10, were rapidly induced in M(LPS) and M(Curdlan) but returned to near basal expression by the 24-h time point. Second, although many genes were similarly regulated in M(IFNβ) and M(IFNγ), the expression patterns of CD163 and CXCL9 were distinct in response to these two interferon types. Third, most markers have been noted because of their increased expression in response to macrophage activation conditions but many transcripts in this panel showed a remarkable reduction in expression. Finally, the expression level of many activation marker transcripts was either continuing to change or was sustained at high levels at the 24-h time point. Together, these observations revealed there is a need for a systematic attempt to identify reliable activation markers whose expression was either up- or down-regulated in human macrophages, and which exhibited sustained expression level changes in response to an array of activation conditions.

### Expression profiling of a diverse array of activated human macrophages

To screen for transcripts representing putative human macrophage activation markers, microarrays were performed using samples collected from human MDMs derived from a single donor, subjected to 33 unique activating conditions (Table 1) for 24 h. Sixteen of the conditions were composed of a single activating stimulus. Eight cytokines comprised the largest category of macrophage-activating stimuli used in this study and represent a spectrum of pro- and anti-inflammatory molecules that are abundantly expressed in sites where MDMs would be recruited such as infections or wounds. Pathogen-associated molecular patterns (PAMPs) recognized by C-type lectin receptors (CLRs) or toll-like receptors (TLRs) were the second largest set of macrophage-activating stimuli in this study and consisted of Curdlan (dectin-1 agonist), TDB (trehalose-6,6-dibehenate; mincle agonist), polyI:C (TLR3 agonist), or one of two concentrations of LPS (TLR4 agonist). Another set of stimuli, IgG–OVA IC and adenosine, were selected for their ability to reprogram inflammatory macrophages to become non-inflammatory (34, 35). Finally, we selected the glucocorticoid, dexamethasone, as an immunosuppressive stimulus. The remaining 17 conditions consisted of pairs of the above macrophage-activating stimuli (Table 1). The macrophage-activating conditions were selected with the expectation that they would lead to diverse gene expression profiles providing insights into the potential diversity of macrophage gene expression programs.

**Figure 2:**
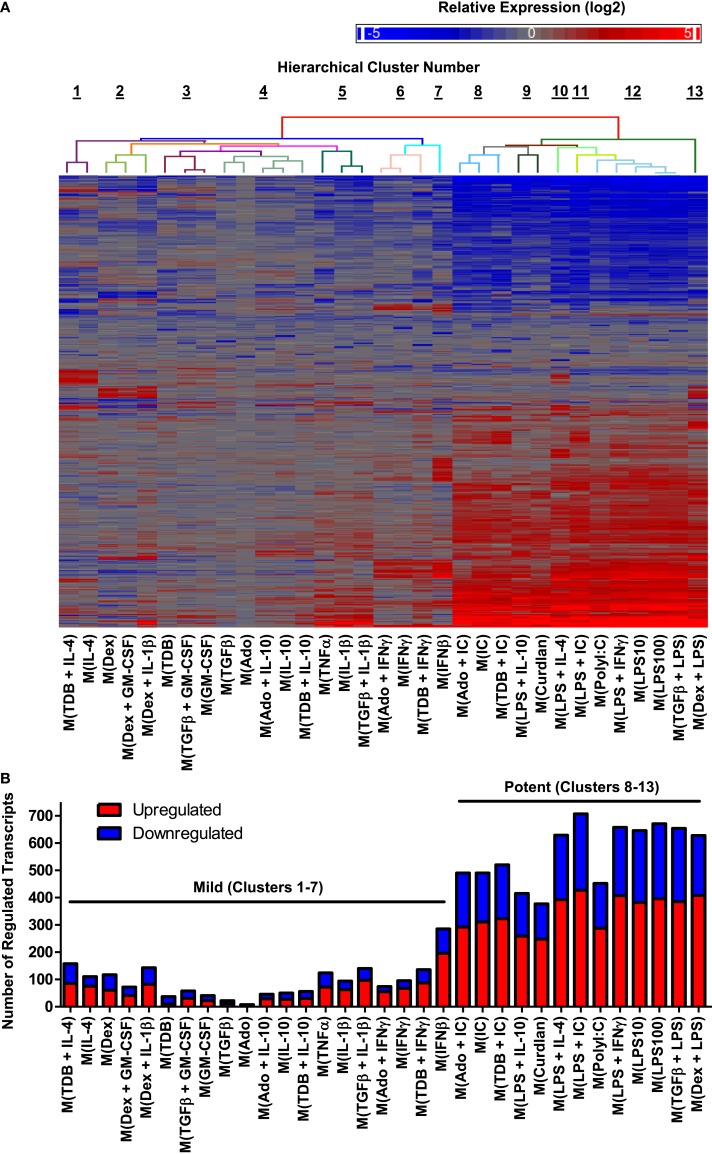
**Hierarchical clustering of gene expression profiles from activated human MDMs separated into 2 major clusters and 13 minor clusters**. Microarrays were performed using RNA collected from MDMs at 24 h post-treatment with 33 distinct activation conditions (*N* = 1). **(A)** A set of 1874 regulated transcripts defined as having >4-fold change in expression levels relative to untreated controls was compiled and displayed as a heat map (log_2_ scale). Gene expression profiles were sorted according to unsupervised hierarchical clustering of genes and treatments. Dissimilarity distances between gene expression profiles are displayed using a color-coded dendrogram to indicate 13 hierarchical clusters. See Section “Results” for dissimilarity distance cut-off rationale. Arranged in the same order as shown here, transcript names and quantitation of expression level changes are available in Table S1 in Supplementary Material. **(B)** Number of upregulated and down-regulated transcripts within each gene expression profile. Potent and mild macrophage activation conditions are indicated.

We first focused our attention on regulated transcripts that had changes in abundance of over fourfold relative to untreated controls changes. A data set of 1874 regulated transcripts that were differentially expressed in MDMs responding to one or more of the macrophage-activating conditions was compiled. Unsupervised hierarchical clustering was performed to evaluate the expression profiles of the regulated transcripts; this is summarized in a heat map that includes a dendrogram indicating relative dissimilarity distances between gene expression profiles of each polarized macrophage type (Figure 2A). Official gene names of the regulated transcripts and calculated expression changes are provided as supplemental material (Table S1 in Supplementary Material).

We considered whether the clustering analysis results separated the gene expression profiles corresponding to previously studied macrophage activation states as denoted in Table 1. Consistent with the previous reports (29, 33), gene expression profiles of M(LPS + IFNγ) (previously named “M1”) macrophages were quite different from that of M(IL-4) (previously named “M2a”) macrophages. By contrast, the profile of M(LPS + IC) macrophages (previously named “M2b”) was very similar to the profiles of M(LPS + IFNγ), separated only by the profile of M(PolyI:C). Since M(LPS + IFNγ) and M(LPS + IC) are known to have different biological activities (6), we divided the 33 macrophage expression profiles into 13 clusters, the lowest dissimilarity distance cut-off that successfully separated these profiles (Figure 2A).

### Microarray results were confirmed using IFC arrays

The IFC array-based real-time RT-PCR platform provided a high-throughput mechanism to accurately verify the expression of a large set of transcripts in samples from multiple human donors. We used several strategies to select a panel of transcripts with diverse expression patterns out of the 1874 regulated transcripts, which were re-assessed on multiple samples using IFC arrays. First, we included the 11 transcripts analyzed in Figure 1. Next, we used the STRING database (version 9.05) to identify enriched GOs for the 1615 protein coding RNAs in our set of 1874 regulated transcripts. Among the GOs categories that were enriched in our data set, we chose to focus on chemokine activity, cell surface, and cytokine activity because these GO categories were highly enriched (Table S2 in Supplementary Material). Finally, we selected transcripts that were uniquely regulated in one or two minor clusters. The final panel included a combination of transcripts that represented changes occurring in each of the 33 macrophage-activating conditions. We also mined the data set for reliable endogenous controls to include in the panel. Among the potential endogenous controls we considered, the expression levels of TBP (define) and B2M (define) transcripts appeared to be the least affected by the 33 macrophage-activating conditions (Table S1 in Supplementary Material).

The samples obtained from activated MDMs of a single donor that were analyzed by microarray were re-assessed using IFC arrays. Approximately 10 transcripts were not detected when using a pre-established Ct cut-off. Strong linear correlation for 15 representative transcripts was observed when comparing expression levels determined by microarray and by IFC arrays (Figure S1 in Supplementary Material). The remaining detectable transcripts in our panel had expression levels that also showed strong linear correlation when comparing microarray and RT-PCR results (data not shown). Overall, these results confirmed the microarray measurements using an independent approach and provide convincing evidence that IFC arrays was a dependable method for measuring transcript expression.

### Macrophage-activating conditions can be categorized as “mild” or “potent” based on the number of transcripts regulated in response to the stimuli

There was a large range in the number of highly regulated transcripts in each activated macrophage expression profile (Figure 2B). Specifically, the 20 activated macrophage types within clusters 1–7 had relatively few regulated transcripts (93 ± 14), whereas the 13 activated macrophage types within clusters 8–13 had large numbers of regulated transcripts (564 ± 31). We propose that macrophage-activating conditions can be categorized as “mild” or “potent” based on the number of transcripts the treatment alters.

When considering the mild and potent clusters of the unsupervised hierarchical clustering, we noted that the gene expression profiles did not segregate along previously described M1–M2 divisions (Table 1). Polarized macrophage types, M(IFNγ) and M(LPS), which have each been considered “M1” macrophage types sorted into the mild and potent clusters, respectively. Similarly, macrophage types formerly named “M2,” M(IL-4), and M(LPS + IC) were categorized as mild and potent, respectively.

We considered the possibility that the wide discrepancy in the number of transcripts regulated in MDMs responding to mild and potent activating conditions was due to suboptimal concentrations of the “mild” stimulus. To address this, MDMs were treated with each of the 11 single treatment macrophage-activating conditions that were categorized as mild at concentrations ranging from 4-fold higher to 16-fold lower those used in the microarray-based experiments. In general, modest dose responses were observed. In response to the majority of the mild stimuli tested (IFNβ, IFNγ, IL-1β, IL-4, IL-10, and TNFα), the amplitude of change in expression for any given transcript was routinely <4-fold between the lowest and highest concentrations for the activating stimulus tested (Figure S2 in Supplementary Material). This suggests that the window of activity is wide for these stimuli and further suggests that use of higher concentrations of these stimuli would be unlikely to revise their macrophage-activating categorization from “mild” to “potent.”

### Evaluation of correlation coefficients between activated MDM gene expression profiles supports conclusions drawn from hierarchical clustering analyses

**Figure 3:**
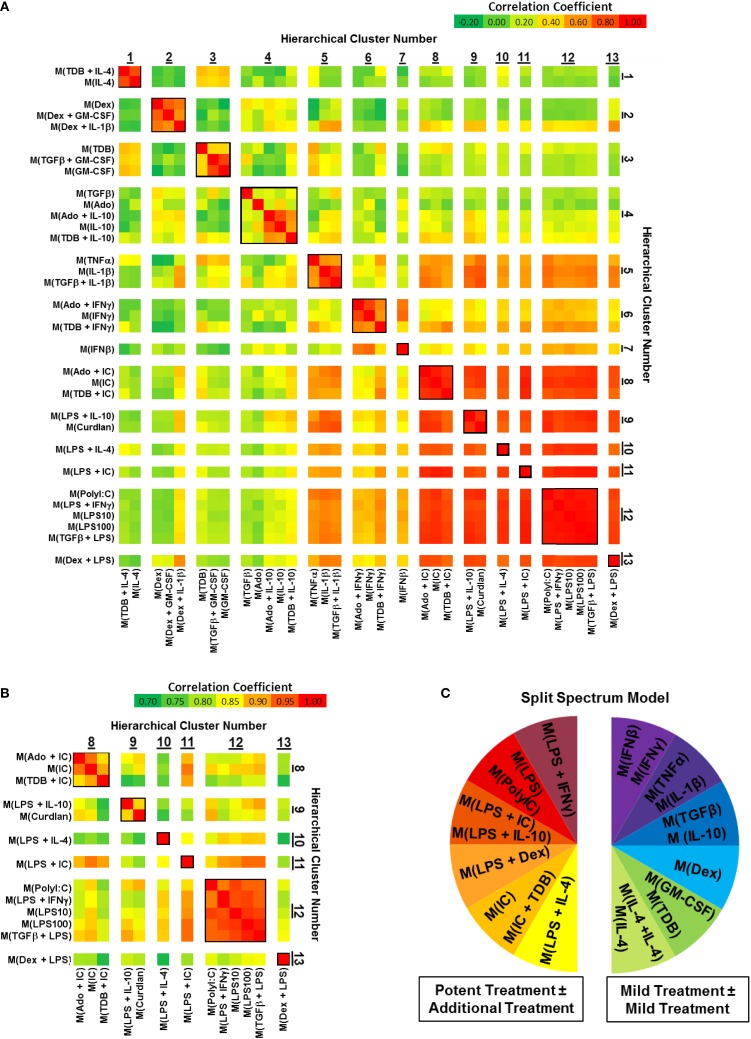
**Comparing correlation coefficients supported the separation of gene expression profiles into two clusters, which can be modeled as a “split spectrum.”** Correlation coefficients were calculated for using the 1874 regulated transcript data set (*N* = 1). **(A)** Each pairwise combination of the 33 gene expression profiles are displayed as a heat map with a range of coefficients of −0.2 to 1.0. **(B)** Each pairwise combination of the 13 “potent” gene expression profiles in clusters 8–13 are displayed as a heat map with a restricted range of coefficients from 0.7 to 1.0. **(C)** A “Split Spectrum” model of macrophage activation can be used to emphasize the high degree of correlation between treatments with at least one potent macrophage-activating stimulus.

Correlation coefficients were determined for each pairwise combination of activated MDM gene expression profiles in the set of 1874 regulated transcripts (Figure 3). This analysis further substantiated the categorization of gene expression profiles into mild and potent categories as shown by unsupervised hierarchical clustering (Figure 2). As an example, there was a consistently higher gene expression profile correlation when the profiles of potently activated macrophages (clusters 8–13) were paired with profiles from potently activated (clusters 1–7), macrophages (Figure 3A). Also, we note that, when using a different color scale (Figure 3B), correlations between profiles in clusters 8–13 were noticeable and supported the division of the gene expression profiles of the potently activated macrophage gene expression profiles into minor clusters.

In a recent microarray-based study (33), at least 9 clusters of activated macrophages in a data set derived from human MDMs activated with 28 distinct stimuli. In agreement with the level of clustering as the previous study, we now show using unsupervised hierarchical clustering and correlation coefficient analyses that human MDMs activated with the 33 macrophage activation conditions used in this study form at least 13 clusters. Both studies support a spectrum model of macrophage activation. Because of the strong “mild” and “potent” macrophage-activating condition categories described here, we propose that macrophage activation may best be described using a “split spectrum” model (Figure 3C).

### Verifying transcripts that serve as markers for the “potent” macrophage activation conditions

**Figure 4:**
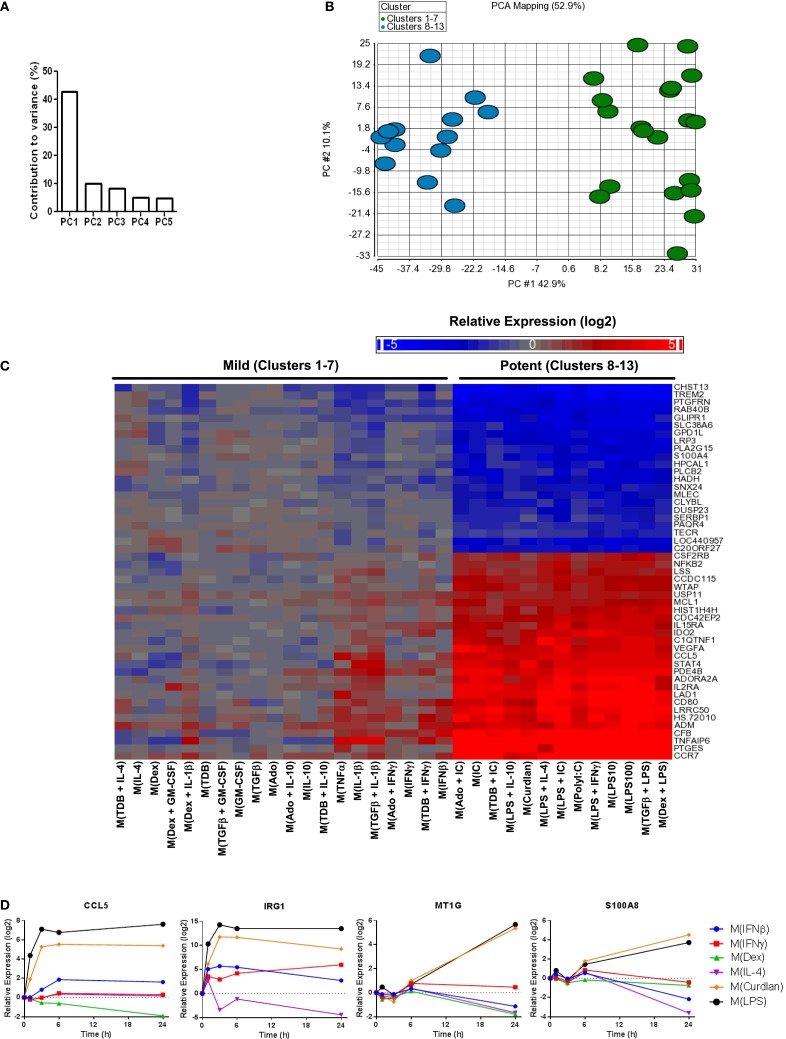
**Transcripts universally regulated by “potent” macrophage-activating conditions in clusters 8–13 were the largest source of variance in the polarized MDM gene expression profiles**. Principal components analysis was performed using the data set of 1874 regulated transcripts from the 33 gene expression profiles. **(A)** The contribution of PC1–PC5 to the variance is shown. **(B)** Scatterplot displays gene expression profiles according to PC1 and PC2 scores with color coding based on “mild” (clusters 1–7) and “potent” (clusters 8–13) categorization. **(C)** The 50 transcripts that contributed the most to PC1 were sorted according to unsupervised hierarchical clustering results. Changes in transcript expression levels relative to untreated MDMs are depicted as a heat map (log_2_ scale). **(D)** Using IFC-based RT-PCR and samples from Figure 1, the changes in expression for each indicated transcript relative to the untreated MDM controls was determined at the 1, 3, 6, and 24 h time points for the six types of activated MDMs (*N* = 1).

The first principal component (PC1) explains (42.9%) of the variance in the data set of 1874 regulated genes while PC2 and PC3 each contributed to ~10% of the variance and the remaining principal components each accounted for <5% of the variance (Figure 4A and data not shown). A scatterplot of regulated gene expression profiles based on the first two principal components segregated profiles in clusters 1–7 from those in clusters 8–13 along the PC1 axis (Figure 4B). The expression profiles of the 50 transcripts that contributed the most to PC1 were subjected to unsupervised hierarchical clustering and displayed as a heat map (Figure 4C). There was an obvious distinction between gene expression responses between the profiles within the mild and potent major clusters; the transcripts robustly regulated by potent macrophage-activating conditions and relatively unaltered by mild macrophage-activating conditions underlie PC1 and account for the major source of variance for the diverse spectrum of polarized macrophage gene expression profiles in this study.

There were many transcripts in addition to the 50 noted in Figure 4C that contributed to PC1. Using samples collected for analysis in Figure 1, we monitored the change in expression of four transcripts (CCL5, IRG1, MT1G, and S100A8) that contributed to PC1 over 24 h (Figure 4D). CCL5 and IRG1 transcripts showed immediate increased expression levels that were sustained through the 24-h time point. By contrast, delayed increases were seen for the expression levels of MT1G and S100A8 transcripts. These four transcripts, in addition to IL1B, IL6, and IL23A that were previously seen to have sustained high expression levels in M(LPS) and M(Curdlan) macrophages, suggesting that numerous transcripts that can be reliably used as markers of “potent” activation conditions.

### Evaluating the use of chemokine transcripts as macrophage activation markers

**Figure 5:**
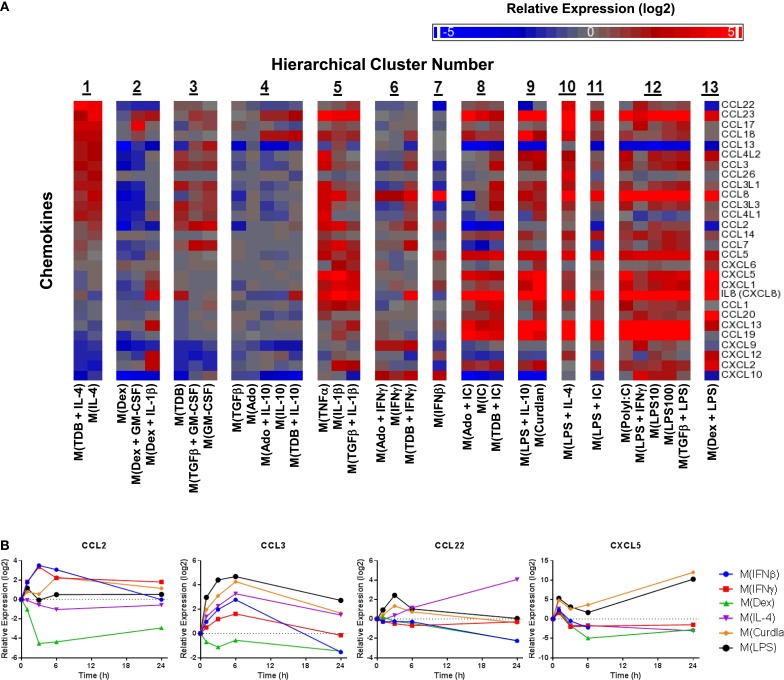
**Evaluation of chemokines as MDM activation markers**. **(A)** All C–C and C–X–C chemokines were selected from the set of 1874 regulated transcripts and sorted according to average expression level changes in response to the two macrophage-activation treatment conditions within cluster 1. **(B)** Using IFC-based RT-PCR and samples from Figure 1, the changes in expression for each indicated transcript relative to the untreated MDM controls was determined at the 1, 3, 6, and 24 h time points for the six types of activated MDMs (*N* = 1).

Chemokines not only play an important functional role in macrophage activity but also include some of the earliest proposed markers of macrophage polarization (2, 5). We generated a heat map of transcript expression changes for chemokines from the C–C and C–X–C subfamilies from the 1874 transcripts (Figure 5A). Since IL-4 treated macrophages have been well characterized, the chemokines were sorted according to their average expression in the two activated macrophage types that form cluster 1, M(IL-4) and M(TDB + IL-4). Among the remaining 12 clusters, the chemokine expression profiles from macrophages in cluster 3, comprised of M(TDB), M(TGFβ + GM-CSF), and M(GM-CSF) macrophages, appeared to have the most similar trend in chemokine expression. The overall chemokine expression patterns from all other profiles shared little resemblance to those in cluster 1.

Transcripts for two chemokines, CCL13 and CCL22, accumulated in macrophages treated with IL-4 for 24 h (Figure 5A). Interestingly, the upregulation of these chemokines in response to IL-4 was delayed relative to other treatments that induced transient upregulation: interferons for CCL13 (Figure 1) and PAMPs for CCL22 (Figure 5B). These observations suggests that CCL13 and CCL22 can be used as specific markers for M(IL-4) as long as enough time has elapsed since the activation occurred.

We noted that nearly all chemokines had reduced expression in M(Dex) macrophages according to the microarray results (Figure 5A). This observation was confirmed when monitoring the kinetics of expression for five chemokine transcripts described above (CCL5, CCL13, CCL18, CXCL9, and CXCL10) (Figures 1 and 4D) and in four additional chemokines (CCL2, CCL3, CCL22, and CXCL5) (Figure 5B). The general trend of repressing chemokine production in M(Dex) macrophages may hint at a mechanism by which dexamethasone acts as an immunosuppressive molecule.

### Donor-to-donor variability in gene expression regulation was minimal in most circumstances but was occasionally seen in some minor clusters

**Figure 6:**
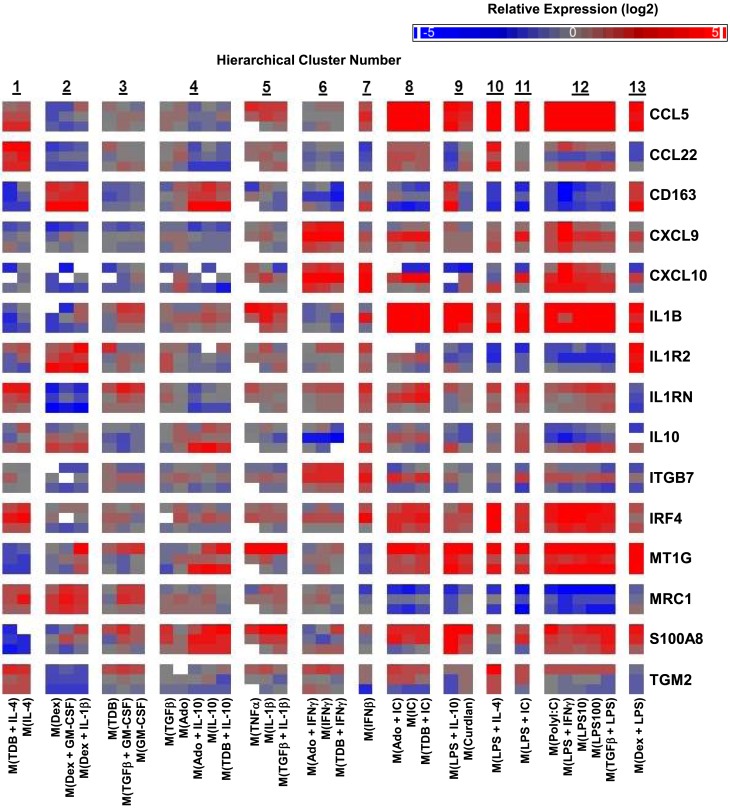
**Variability in donor-to-donor MDM gene expression responses was often limited to specific clusters**. IFC-based RT-PCR was used to determine the expression of 48 transcripts (45 putative macrophage activation markers and 3 endogenous controls) in MDMs at 24 h post-treatment with 33 distinct activation conditions (columns) (*N* = 3). Shown here are the results for 15 of the activation marker transcripts. The RNA collected from the first donor (first row for each indicated transcript) had been used in the microarray studies and the RNA from two additional donors (second and third row for each indicated transcript) was collected in independent experiments. Blank areas within clusters represent samples did not meet the Ct cut-off of 25 or, in the case of the third M(TNFα) sample, did not load properly into the IFC device.

A caveat to the results described until this point is that they were based on MDMs derived from two human donors: one donor for monitoring transcript expression kinetics and one donor for transcriptional profiling. Since donor-to-donor variability among human MDM responses was a concern, the expression profiles for many transcripts was determined in samples derived from the microarray experiment and from two additional donors whose MDMs were treated with all 33 macrophage activation conditions (Figure 6).

The strong correlation between the microarray results and the IFC PCR results for the first donor was discussed above (Figure S1 in Supplementary Material). Importantly, the last two rows for each transcript, which show the results for the two additional donors, indicated that the MDM responses were, in general, similar to those of the first donor (Figure 6). There were a few transcripts (CCL22, CXCL10, IL10, ITGB7, and TGM2) that had strong opposing changes in expression from one donor to the next (Figure 6). It is noteworthy that in these instances, the difference in expression was restricted to a limited number of clusters. For example, CCL22 expression regulation tended to be similar in response to all 33 macrophages activation conditions for all 3 donors; the notable exception was seen in the 5 macrophages activation conditions within cluster 12 for the second donor (Figure 6). This result is unlikely due to the polyIC and LPS treatments being suboptimal in the experiment involving MDMs from donor 2 since other transcripts, such as CCL5, were regulated similarly in all three donors for the macrophage-activating conditions that make up cluster 12.

**Figure 7:**
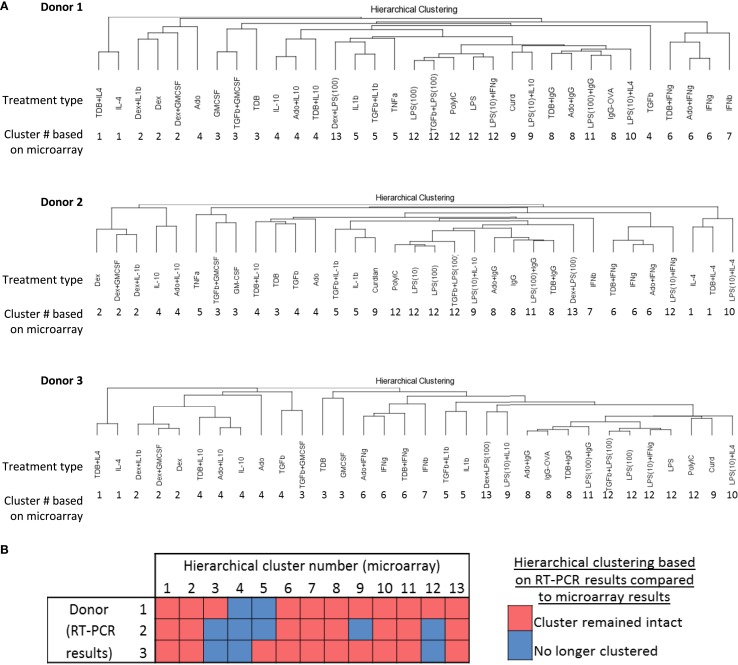
**Comparing unsupervised hierarchical clustering of 33 activated macrophage types based on 1874 regulated transcripts against hierarchical clustering based on a 45-transcript subset of putative activation markers**. IFC PCR was used to determine the expression of 48 transcripts in MDMs at 24 h post-treatment with 33 distinct activation conditions (columns) (*N* = 3). The RNA collected from the first donor had been used in the microarray studies and the RNA from two additional donors was collected in independent experiments. Data points were omitted when the ΔCt value was unreliable as defined by either the macrophage activation marker or the endogenous control not meeting the Ct cut-off of 25. **(A)** Unsupervised hierarchical clustering was performed using calculated ΔΔCt values derived from IFC PCR. Dissimilarity distances between gene expression profiles are displayed as dendrograms for each donor. For comparison purposes, the hierarchical cluster number is displayed below each macrophage-activating treatment type. **(B)** A summary is shown for comparisons between microarray-derived clusters from donor 1 and IFC PCR-derived clusters from donors 1, 2, and 3.

A recent mass cytometry-based study produced a high-dimension data set from a panel of 38 antibodies to effectively identify signature expression patterns of myeloid cell populations in mice from a number of tissues (36). Since the dimensionality of data sets produced by mass cytometry and IFC PCR are similar, we tested whether the 13 clusters originally defined by unsupervised hierarchical clustering of the 1874 regulated transcripts (Figure 2) could be effectively identified using IFC PCR results (Figure 7A). The majority of the 13 clusters remained clusters for each of the three donors (Figure 7B). Even the “clusters” composed of a single type of activated macrophage type [i.e., M(IFNβ)] maintained their distinctness relative to the other activated macrophage types. We conclude that gene expression platforms such as IFC PCR monitor a large enough set of macrophage activation marker transcripts to identify an overall macrophage population’s type/cluster while still allowing for detection of subtle donor-to-donor differences.

### Putative activation markers were identified for specific clusters of polarized human macrophages

**Figure 8:**
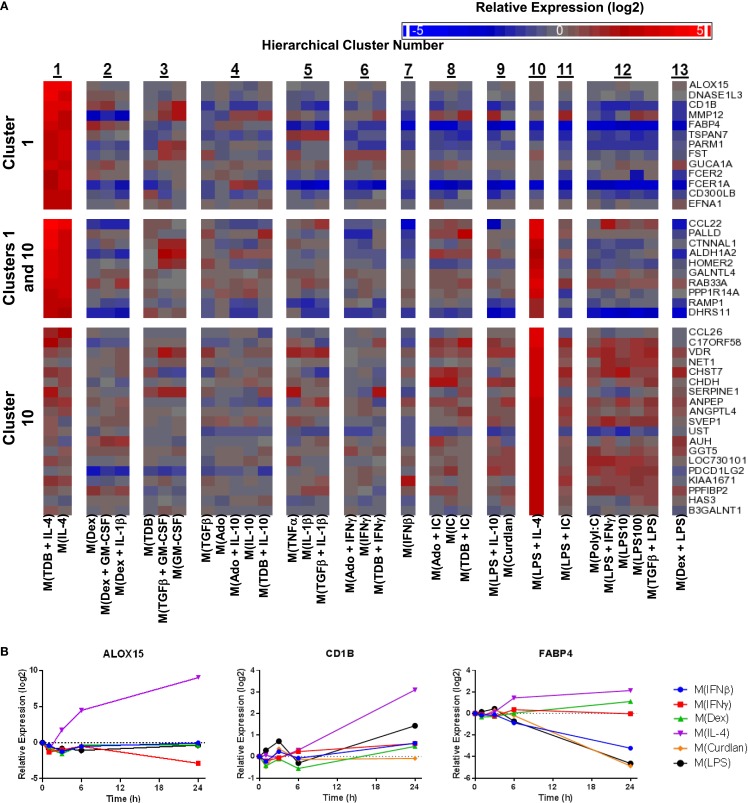
**Evaluation of activation markers in MDMs responding to treatments with IL-4**. **(A)** Putative macrophage activation markers were screened for within the microarray data that met two criteria: (i) a >4-fold expression level change in response to activation conditions that included IL-4 (samples within cluster 1 and/or cluster 10) relative to untreated MDMs and (ii) a >2-fold expression level change relative to the activating conditions that did not include IL-4. **(A)** Changes in select putative activation markers as determined by microarray analysis are shown as a heat map (log_2_ scale) (*N* = 1) **(B)** Using IFC-based RT-PCR and samples from Figure 1, the changes in expression for each indicated transcript relative to the untreated MDM controls was determined at the 1, 3, 6, and 24 h time points for the six types of activated MDMs (*N* = 1).

**Figure 9:**
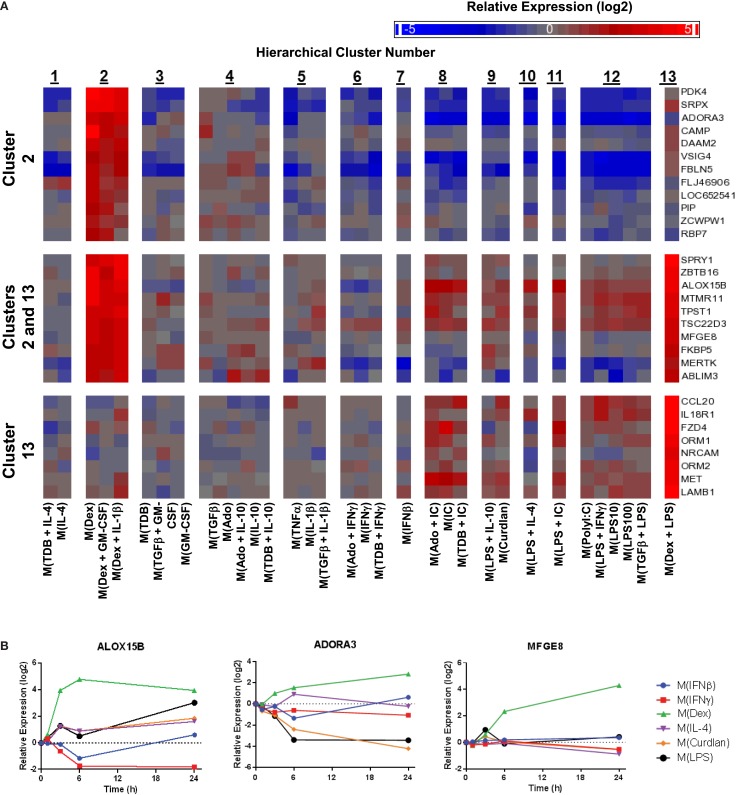
**Evaluation of activation markers in MDMs responding to treatments with dexamethasone**. **(A)** Putative macrophage activation markers were screened for within the microarray data that met similar criteria as described in Figure 7A with a focus on transcripts that changed in response dexamethasone treatment (samples within clusters 2 and/or 13). **(B)** Using IFC-based RT-PCR and samples from Figure 1, the changes in expression for each indicated transcript relative to the untreated MDM controls was determined at the 1, 3, 6, and 24 h time points for the six types of activated MDMs (*N* = 1).

**Figure 10:**
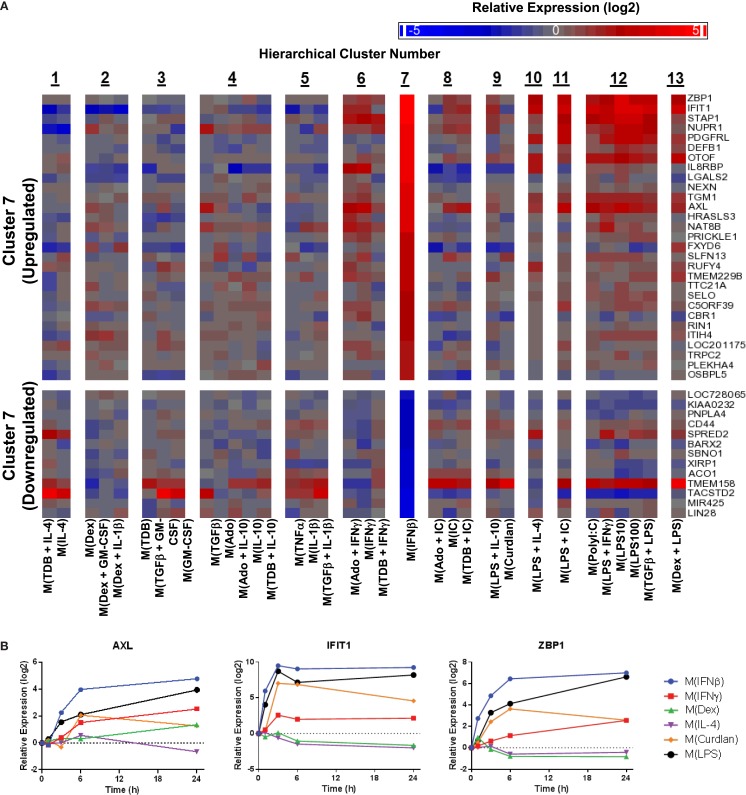
**Evaluation of activation markers in MDMs responding to treatment with IFNβ**. **(A)** Putative macrophage activation markers were screened for within the microarray data that met similar criteria as described in Figure 7A with a focus on transcripts that changed in response IFNβ treatment (cluster 7). **(B)** Using IFC-based RT-PCR and samples from Figure 1, the changes in expression for each indicated transcript relative to the untreated MDM controls was determined at the 1, 3, 6, and 24 h time points for the six types of activated MDMs (*N* = 1).

Macrophage activation markers would ideally have large expression changes in a single cluster or polarized macrophage type. We therefore queried the gene expression profiles in the current study to identify activation markers specific to each of the 13 clusters formed by the unsupervised hierarchical clustering analysis. Many putative activation markers were identified in macrophages activated with IL-4, dexamethasone, or IFNβ (Figures 8–10).

IL-4 was used as an activation condition for gene expression profiles in clusters 1 and 10. We identified transcripts that were strongly upregulated only within cluster 1, within both cluster 1 and cluster 10, or only within cluster 10. Examples of transcripts that fit these gene expression profiles were readily detected within our data set (Figure 8A). Analysis of the kinetics of expression for three of the transcripts identified by this screening approach showed that while ALOX15 and CD1B each appear to be good markers for M(IL-4), although the increase in CD1B was delayed until the 24 h time point, FABP4 was not robustly induce in M(IL-4) but could still be a valuable marker as this transcript was potently down-regulated in response to several macrophage-activating conditions (Figure 8B). This latter observation was consistent with the microarray data (Figure 8A).

Given the relative ease of finding IL-4-associated activation markers in our data set, we switched our attention to identifying additional activation markers. Dexamethasone-associated activation markers were identified that were specifically upregulated in macrophage-activating conditions from only within cluster 2, within both clusters 2 and 13, and only within cluster 13 (Figure 9A). The expression kinetics was determined for three of the transcripts identified by the microarray screen as dexamethasone responsive (Figure 9B). Of these, ALOX15B and MFGE8 appear to be a markers for M(Dex) at early and late time points, respectively.

Next, potential activation markers or IFNβ-treated macrophages were identified within cluster 7 (Figure 10A). Further analysis showed that AXL, IFIT, and ZBP1 were all induced rapidly in M(IFNβ) and with delayed kinetics in M(LPS) (Figure 10B). This observation may be explained by indirect induction of these genes by LPS-induced IFNβ production.

## Discussion

Characterization of TAMs has shifted from quantifying macrophage density in and around tumors to evaluating markers of activation (15, 37). It is important to note that macrophage activation markers have been used to categorize macrophage activation, typically using the M1–M2 nomenclature, yet the regulation patterns of these markers in macrophages responding to a wide variety of activation conditions are not well understood. Using a combined microarray- and IFC array-based approach in this study, previously proposed markers of macrophage activation were better characterized and novel markers of macrophage activation were identified.

In the earliest report using M1–M2 nomenclature, the authors stated that “M-1 and M-2, while useful for conceptualizing immune responses, certainly could be an oversimplification” and that “there may be a continuum of phenotypes between M-1 and M-2 macrophages” (3). A recently proposed framework argued against using the M1–M2 nomenclature yet upheld the linear model concept that suggested M(IFNγ) and M(IL-4) to represent the polar extremes (9). However, both the results of the current study and those reported by Xue et al. (33) support a spectrum model of macrophage activation rather than a linear model.

Unsupervised hierarchical clustering, correlation coefficient analysis, and principal components analysis of the regulated transcripts each support the concept that macrophage polarized states in this study can be sorted into two major clusters. We designated these clusters “mild” and “potent” to convey the number transcripts altered in response to each specific macrophage-activating condition. It is important to note that, although we have evaluated more macrophage activation conditions in a macrophage activation study that has previously been published, there could be activation conditions that will have an intermediate number of regulated transcripts making our split spectrum model potentially incorrect. Indeed, Xue et al. (33) studied macrophage responses to 28 activation conditions and we found that the free fatty acid conditions from their study may represent an “intermediate” cluster (analysis not shown). While our “split spectrum” model may not represent the entirety of the spectrum, it raises the idea that strength of macrophage activation may be worth considering in future attempts to accurately describe macrophage activation/polarization.

In the analysis of the principal components, special attention was warranted for PC1 because it accounted for four times more of the variance than any other principal component. The single treatment macrophage-activating conditions that contributed to PC1 were immune complexes, Curdlan, polyIC, and LPS. All combinational treatments that contributed to PC1 contained one or two of these potent stimuli. Treatment of macrophages with immune complexes and Curdlan initially signal through Fcγ receptor/Syk/Card9 pathways while treatment with polyIC and LPS signal through TRIF and/or MyD88 pathways. Despite these initial differences, there is substantial overlap triggered by the potent stimuli further downstream pathway signaling. For example, activation of pathways such as NF-κB and MAPK may directly and indirectly account for the regulated expression of many transcripts that contributed to PC1. Importantly, as noted in Table 1, chicken ovalbumin and Curdlan are often contaminated with substantial levels of endotoxin, so the “potent” activation conditions may be mostly or in part a consequence of TLR-initiated signaling (10, 11). Future studies will assess the extent that TLR signaling may have contributed to the alterations in the M(Curdlan) and M(IC) macrophage gene expression profiles.

There was substantial evidence, both gene expression and functional, that the mild and potent polarized macrophage types of our data set should be divided into smaller clusters. To define these clusters, we chose to separate our gene expression profiles based on known differences that occur in response macrophage-activating conditions rather than using a statistically based dissimilarity cut-off in the unsupervised hierarchical clustering. Specifically, we noted that M(LPS + IFNγ) and M(LPS + IC) were situated close to each other according to unsupervised hierarchical clustering analysis (Figure 2). Important functional differences in macrophages treated with these two distinct activating conditions such as cytokine production (IL-12 vs. IL-10) and ability to skew CD4^+^ T cell responses (Th1 vs. Th2) (35, 38–40) supported the segregation of these gene expression profiles into separate clusters. Therefore, the dissimilarity distance between these two gene expression profiles served as our cut-off to rationally sort the 33 gene expression profiles into 13 clusters.

It is notable that if the gene expression profiles had been segregated based on dissimilarity distances into 14 clusters instead of 13, the 5 gene expression profiles currently grouped within “cluster 4” would have been split into 2 clusters. Furthermore, correlation coefficients within cluster 4 were markedly higher when comparing gene expression profiles from MDMs activated with conditions that included IL-10 (Figure 3). Finally, hierarchical clustering based on IFC PCR results (Figure 7) failed to retain the integrity of cluster 4 in any of the three donors. These observations suggest that subdividing the 33 gene expression profiles into more than 13 clusters may have been warranted starting with subdividing cluster 4. Future functional studies will be useful for supporting or modifying our current classification of 13 clusters for these 33 macrophage-activating conditions.

In order for macrophage activation markers to be useful, it is critical to know whether each marker is regulated by a wide variety or a limited number of stimuli. In our initial time course analysis survey of previously proposed macrophage activation markers, few of the 11 transcripts were found to be highly specific for a specific type of activated macrophage. Therefore, microarrays were performed and then surveyed to identify novel macrophage activation markers. This approach proved to be useful for identifying markers differentially expressed by activated macrophages in all the potent conditions used in this study (Figure 4) and in many of the minor clusters (Figures 7–9).

Our approach of screening for macrophage activation markers by surveying microarray results of a single donor’s macrophage responses to 33 different activation conditions and following up with IFC arrays proved effective. Also, use of unbiased, bottom-up analyses of the microarray results argue against previously proposed top-down linear frameworks describing macrophage activation states, such as the M1–M2 system (3, 9). We note that our results are in line with the spectrum model proposed by Xue et al. from their microarray data set (33). There are likely to be more clusters of activated macrophages than the 13 described here and the 9 described by Xue et al. (33). Taken together, we conclude that measuring the expression changes in a panel of well-characterized markers would provide a useful tool to accurately differentiate various activation states associated with functional activity of TAMs or other macrophage populations.

## Conflict of Interest Statement

The authors declare that the research was conducted in the absence of any commercial or financial relationships that could be construed as a potential conflict of interest.

## Supplementary Material

The Supplementary Material for this article can be found online at http://journal.frontiersin.org/article/10.3389/fimmu.2015.00253

Table S1 **Gene expression profiles in the 33 activated macrophage types for the 1874 regulated transcripts**.

Table S2 **GO enrichment for 1615 gene names recognized by STRING v. 9.05**.

Figure S1 **IFC PCR-calculated transcript expression changes correlated well with results from the microarrays**. Scatterplots of gene expression level changes of the indicated transcripts as determined by microarray and by Fluidigm IFC-based RT-PCR. RNA samples collected at 24 h post-treatment from activated MDMs of a single donor were used as template for both assays.

Figure S2 **Dose-dependent changes in MDM transcript expression levels were minimal across a broad range of concentrations for most of the mild, single stimulus treatments. IFC-based RT-PCR was used to monitor the expression of 48 transcripts in MDMs from a single donor treated with four different concentrations of 11 indicated mild treatment stimuli**. The concentrations tested were 4×, 1×, 1/4×, and 1/16× relative to the concentration described for each stimulus in Table 1. For each treatment, transcripts that had at least a fourfold change in expression (>2 or <−2 on log_2_ scale) in any of the four tested concentrations were selected for display.
