# Supplementary figures and images for: A Systematic Approach to Identify Markers of Distinctly Activated Human Macrophages
Source: Front Immunol. 2015 May 27;6:253. doi: 10.3389/fimmu.2015.00253 (PMC4445387; doi:10.3389/fimmu.2015.00253)

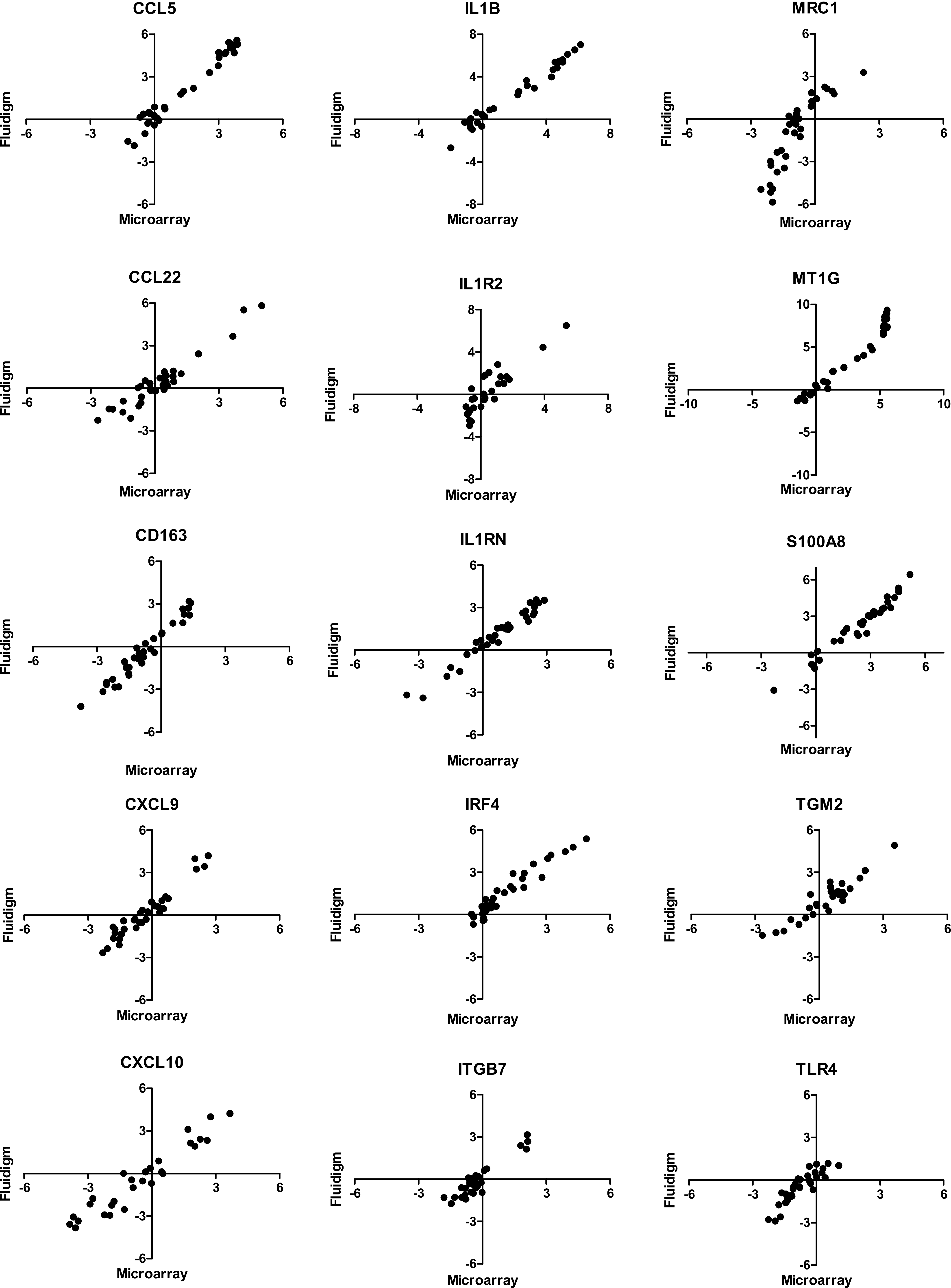

Supplement: Figure S1 — IFC PCR-calculated transcript expression changes correlated well with results from the microarrays. Scatterplots of gene expression level changes of the indicated transcripts as determined by microarray and by Fluidigm IFC-based RT-PCR. RNA samples collected at 24 h post-treatment from activated MDMs of a single donor were used as template for both assays. [file Image_1.TIF]

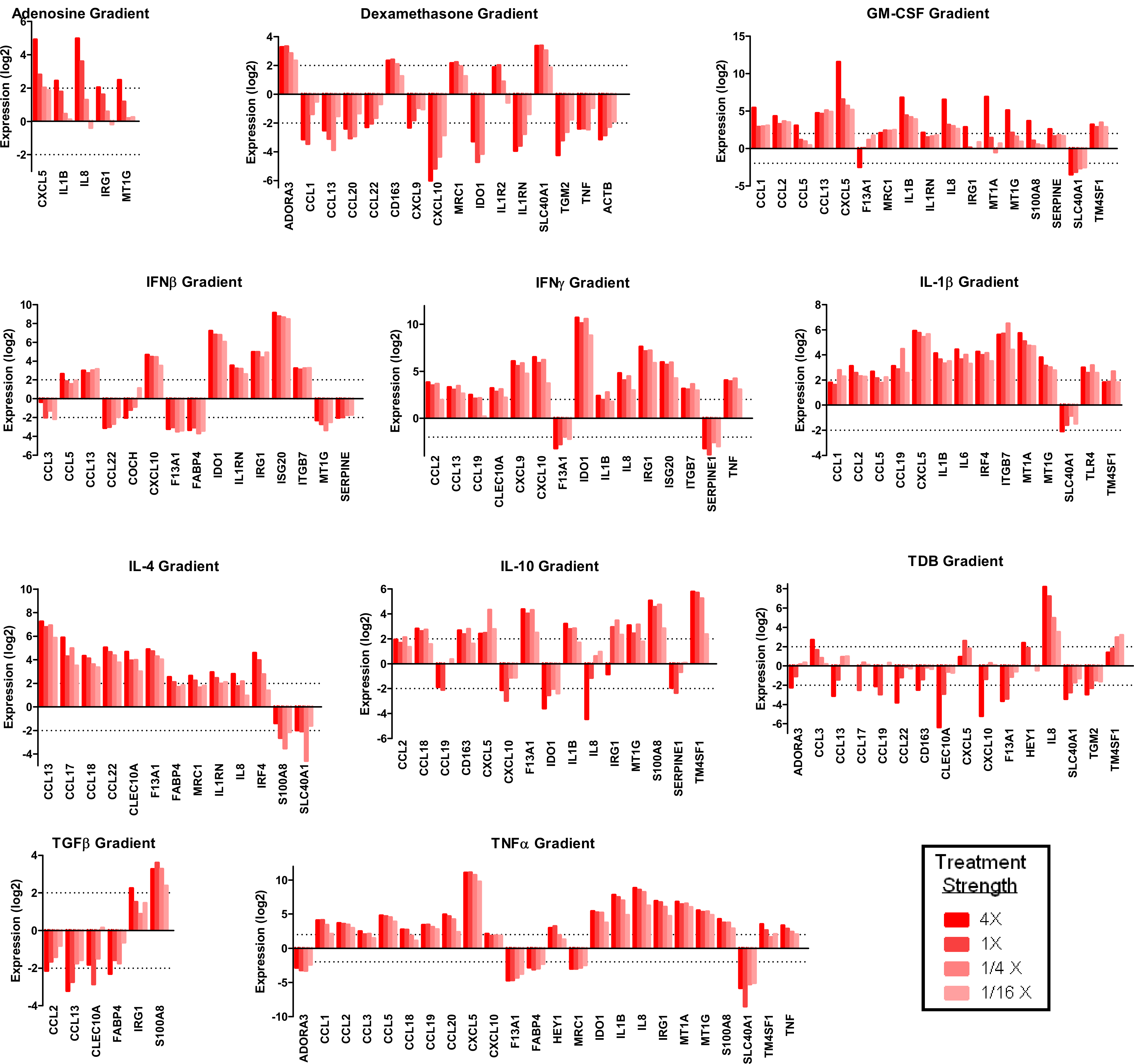

Supplement: Figure S2 — Dose-dependent changes in MDM transcript expression levels were minimal across a broad range of concentrations for most of the mild, single stimulus treatments. IFC-based RT-PCR was used to monitor the expression of 48 transcripts in MDMs from a single donor treated with four different concentrations of 11 indicated mild treatment stimuli. The concentrations tested were 4×, 1×, 1/4×, and 1/16× relative to the concentration described for each stimulus in Table 1. For each treatment, transcripts that had at least a fourfold change in expression (>2 or <−2 on log2 scale) in any of the four tested concentrations were selected for display. [file Image_2.TIF]
